# 291. Duration of Antibiotic Therapy for Uncomplicated Gram-Negative Bacteremia in Solid Organ Transplantation

**DOI:** 10.1093/ofid/ofaf695.094

**Published:** 2026-01-11

**Authors:** William R Cappuccio, Emily L Heil, Mandee Booth, Ashley Barnes, Kimberly C Claeys, Hyunuk Seung, Sara Lee

**Affiliations:** University of Maryland School of Pharmacy, Baltimore, Maryland; University of Maryland School of Pharmacy, Baltimore, Maryland; University of Maryland School of Pharmacy, Baltimore, Maryland; Institute of Human Virology, Baltimore, Maryland; University of Maryland Baltimore, Baltimore, Maryland; University of Maryland School of Pharmacy, Baltimore, Maryland; University of Maryland Medical Center, Baltimore, Maryland

## Abstract

**Background:**

We hypothesize that short durations (≤10 days) of antibiotics for uncomplicated gram-negative bacteremia (GNB) would have similar clinical outcomes compared to long durations ( >10 days) in solid organ transplant (SOT) patients.

DOOR Probability Forest Plot

**Figure 1.**
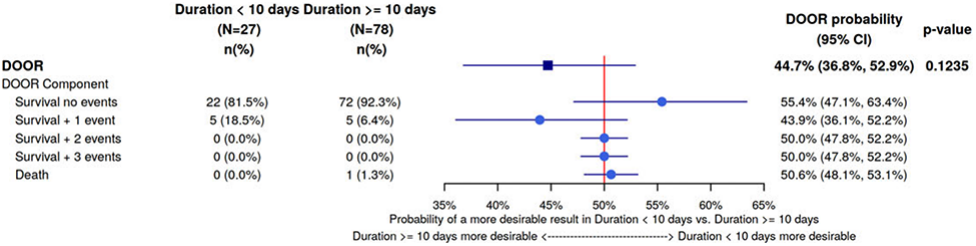
Forest plot demonstrating the desirability of outcome ranking (DOOR) probabilities for each individual DOOR component (survival without adverse events, microbiologic failure, isolation of an MDR gram-negative in the blood, C. difficile infection or death) as well as the overall DOOR probability of having a more desirable outcome with < 10 days versus ≥10 days of antimicrobial treatment.

**Methods:**

A retrospective cohort review was conducted on patients admitted to University of Maryland Medical System hospitals from 1/1/2019 to 6/30/2024. Included patients were ≥18 years old with an uncomplicated GNB defined as a confirmed Enterobacterales bacteremia, source control, and clinical stability at 72 hours. The primary outcome was a Desirability of Outcome Ranking (DOOR) comparison between < 10 days versus ≥10 days of treatment. Each patient was assigned a DOOR rank from 1 (survival without adverse events) to 5 (death). Ranks 2-4 included patients who survived but had 1, 2, or 3 undesirable events occur during the 30-day follow-up period, respectively. Undesirable events included microbiologic failure, isolation of an MDR gram-negative in the blood, or *C. difficile* infection. Confidence intervals for the DOOR probability were calculated using the Halperin et al. (1989) method with a 95% confidence level, with a statistically significant two-sided p-value of < 0.05.

**Results:**

A total of 105 patients were included in this study, with 27 patients in < 10 day cohort and 78 patients in the ≥10 day cohort. The source of GNB was significantly different between groups (p=0.007), with the < 10 day cohort having more intrabdominal infections (48.2% vs 19.2%) and the ≥ 10 cohort having more urinary sources of infection (67.9% vs 37.0%). There were more patients with an oral switch in the ≥10 day treatment cohort (66.7% vs 33.3%, p=0.003). The overall DOOR probability of having a more favorable outcome with a treatment duration of < 10 days compared to ≥10 day treatment was not statistically different (44.7%, 95% CI: 36.8%, 52.9%; p = 0.1235), highlighting that longer durations of therapy were not associated with improved outcomes.

**Conclusion:**

In patients with a solid organ transplant and uncomplicated GNB, there was no significant difference in outcomes when treated for < 10 days or ≥ 10 days. Further studies with larger sample sizes are needed to highlight the efficacy and safety of shorter antibiotic durations for uncomplicated GNB in solid organ transplantation.

**Disclosures:**

Emily L. Heil, PharmD, MS, Wolters-Kluwer: Advisor/Consultant

